# Supplementary material for: Causal Relationship between Aging and Anorexia Nervosa: A White-Matter-Microstructure-Mediated Mendelian Randomization Analysis
Source: Biomedicines. 2024 Aug 16;12(8):1874. doi: 10.3390/biomedicines12081874 (PMC11351342; doi:10.3390/biomedicines12081874)
Supplement: Supplementary file 1 [file biomedicines-12-01874-s001.zip › Supplementary Figure/Figure S4/Singe SNP plot of MR analysis for TL to ubm-b-1616.pdf]

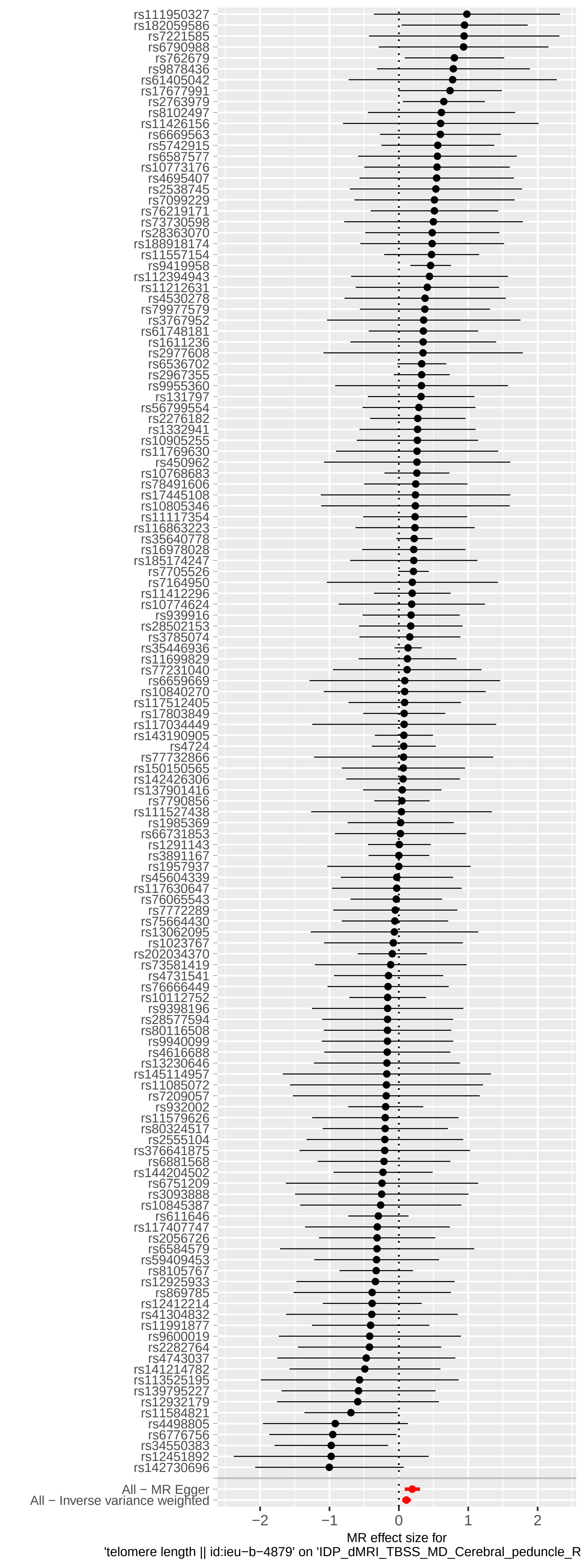
rs111950327
rs182059586
rs7221585
rs6790988
rs762679
rs9878436
rs61405042
rs17677991
rs2763979
rs8102497
rs11426156
rs6669563
rs5742915
rs6587577
rs10773176
rs4695407
rs2538745
rs7099229
rs76219171
rs73730598
rs28363070
rs188918174
rs11557154
rs9419958
rs112394943
rs11212631
rs4530278
rs79977579
rs3767952
rs61748181
rs1611236
rs2977608
rs6536702
rs2967355
rs9955360
rs131797
rs56799554
rs2276182
rs1332941
rs10905255
rs11769630
rs450962
rs10768683
rs78491606
rs17445108
rs10805346
rs11117354
rs116863223
rs35640778
rs16978028
rs185174247
rs7705526
rs7164950
rs11412296
rs10774624
rs939916
rs28502153
rs3785074
rs35446936
rs11699829
rs77231040
rs6659669
rs10840270
rs117512405
rs17803849
rs117034449
rs143190905
rs4724
rs77732866
rs150150565
rs142426306
rs137901416
rs7790856
rs111527438
rs1985369
rs66731853
rs1291143
rs3891167
rs1957937
rs45604339
rs117630647
rs76065543
rs7772289
rs75664430
rs13062095
rs1023767
rs202034370
rs73581419
rs4731541
rs76666449
rs10112752
rs9398196
rs28577594
rs80116508
rs9940099
rs4616688
rs13230646
rs145114957
rs11085072
rs7209057
rs932002
rs11579626
rs80324517
rs2555104
rs376641875
rs6881568
rs144204502
rs6751209
rs3093888
rs10845387
rs611646
rs117407747
rs2056726
rs6584579
rs59409453
rs8105767
rs12925933
rs869785
rs12412214
rs41304832
rs11991877
rs9600019
rs2282764
rs4743037
rs141214782
rs113525195
rs139795227
rs12932179
rs11584821
rs4498805
rs6776756
rs34550383
rs12451892
rs142730696
All − MR Egger
All − Inverse variance weighted
−2
−1
0
1
2
MR effect size for
'telomere length || id:ieu−b−4879' on 'IDP_dMRI_TBSS_MD_Cerebral_peduncle_R
